# Multimodality Imaging for the Evaluation of an Undifferentiated Pleomorphic Sarcoma Presenting as Cardioembolic Stroke

**DOI:** 10.1155/2022/2749303

**Published:** 2022-03-15

**Authors:** Ricardo O. Escárcega, David Bailey, Michael P. DeFrain

**Affiliations:** ^1^Florida Heart Associates and Heart and Vascular Institute, Lee Health System, Fort Myers, FL, USA; ^2^Shipley Cardiothoracic Center, Lee Health System, Fort Myers, FL, USA

## Abstract

**Background:**

Cancer and ischemic stroke are associated with significant morbidity and mortality. Hypercoagulability, disseminated intravascular coagulation, venous-to-arterial embolism, and non-bacterial thrombotic endocarditis are among recognized mechanisms. Emboli to the brain, or to other organs, are known to occur as a consequence of liberated thrombotic debris originating from the thrombogenic surface of intracardiac neoplastic entities. The most common primary malignancy of the heart is sarcoma; however, masses that occur in the heart are 20 to 40 times more likely as a consequence of metastasis from other sites. *Case Report.* A 67-year-old woman presented to the emergency room with two brief episodes of dizziness and diplopia for 2 minutes. She had a medical history of provoked upper extremity DVT after a fracture, hypothyroidism, hyperlipidemia, and soft tissue sarcoma. The sarcoma was initially diagnosed in, and subsequently resected from, the right triceps muscle. During posttreatment surveillance, a second lesion was discovered in the left upper pulmonary lobe, and this was also completely resected 9 months following initial diagnosis. We present a case of a woman with a tertiary (cardiac) site sarcoma that presented with embolic stroke.

**Conclusion:**

Our case highlights the benefits of multimodality imaging, heart-team approach with oncology support to define anatomy, thereby enable surgical treatment, of a complex intracardiac lesion.

## 1. Introduction

Cardioembolic debris is a well-established source of ischemic stroke that has a robust clinical presence in everyday hospital practice. Of those presenting with ischemic stroke, a cardioembolic source is implicated in 15-20% of the cases [1]. Reports in the literature emphasize the importance of echocardiography determining the possibility of a cardioembolic cause [2, 3], and the most recent stroke guidelines recommend echocardiography in the evaluation of suspected ischemic stroke [4].

Cancer and ischemic stroke are associated with significant morbidity and mortality [5]. Hypercoagulability, disseminated intravascular coagulation, venous-to-arterial embolism, nonbacterial thrombotic endocarditis are among recognized mechanisms [5]. A stroke can also occur due to tumor-related embolism [6, 7]. This cardioembolic debris can consist of neoplastic material itself, or thrombus material that can form on the surface of the neoplasm when it is exposed to the bloodstream [8, 9]. Primary cardiac tumors are rare, but the most common malignant types are the sarcomas [10]. Cardiac sarcomas are also common as metastatic lesions. In one series, cardiac metastases were present in 25% consecutive autopsies in patients diagnosed with soft-tissue sarcoma, and of these, 50% had metastasis to the myocardium, 33% to the pericardium, and 17% both [11]. Metastatic lesions to the heart occur 20 to 40 times more common than primary neoplasms [12]. Metastases to the heart can occur from nearly any source, but the most common are the melanoma and carcinomas of the lung, breast, and esophagus [5]. We present a patient with a tertiary site cardiac sarcoma that experienced an embolic stroke which was successfully treated with complete resection.

## 2. Case Report

A 67-year-old healthy appearing woman experienced dizziness and 2 episodes of brief diplopia leading to an acute inpatient evaluation. The patient had a history of provoked upper extremity deep vein thrombosis (DVT) after a fracture, hypothyroidism, hyperlipidemia, and soft tissue sarcoma of the right triceps. Physical exam, including gross neurologic function, was normal at time of presentation. Laboratory analysis was normal. Magnetic Resonance Imaging (MRI) of the brain demonstrated cerebral hemispheric and cerebellar foci which were felt to represent embolic lesions.

**Figure 1:**
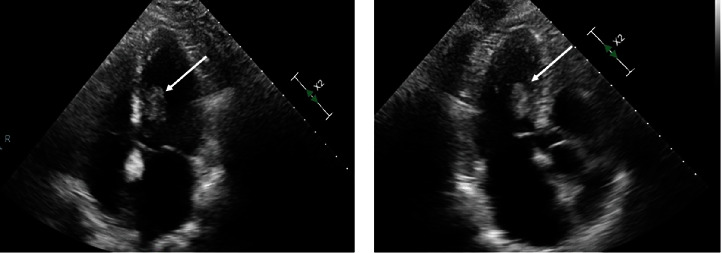
Transthoracic echocardiogram. Panels (a) (apical 4-chamber view) and (b) (apical 3-chamber view) show an echogenic mass located inside the left ventricle attached to the interventricular septum.

**Figure 2:**
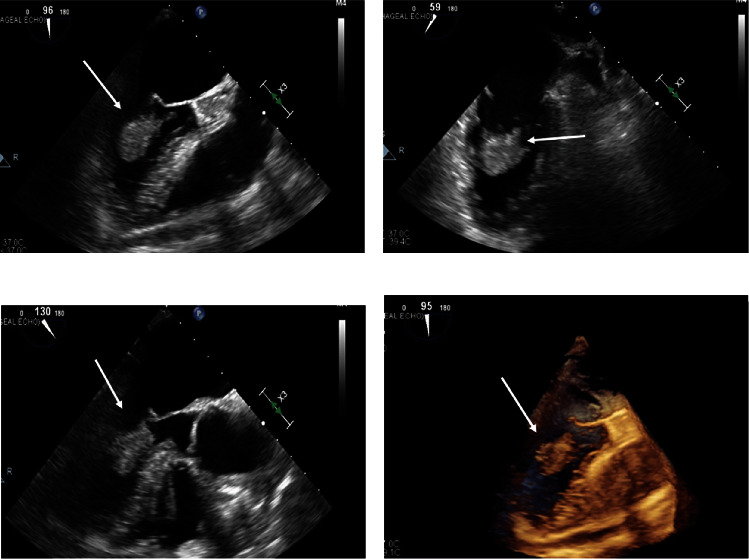
Transesophageal echocardiogram. Panels (a), (b), (c), and (d) show a large echogenic mass attached to the mitral valve apparatus and interventricular septum at different orthogonal views.

**Figure 3:**
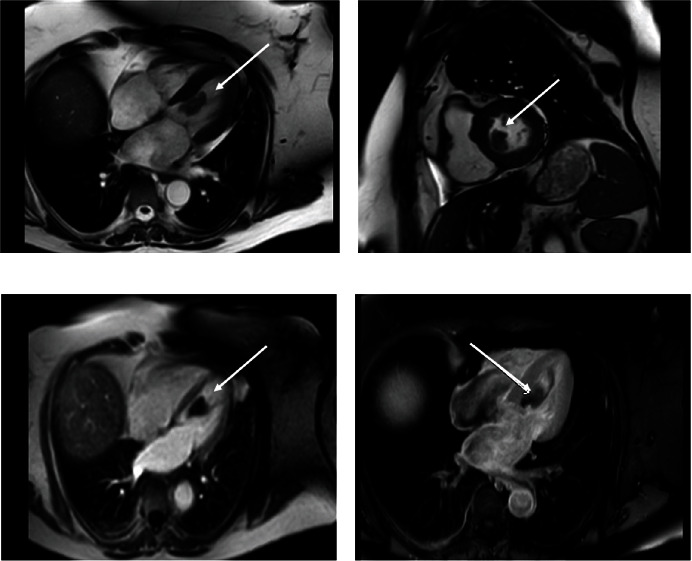
Cardiac MRI. Panels (a), (b), (c), and (d) show in different sequences a mass attached to the mitral valve apparatus and interventricular septum.

18 months prior to presentation, the patient had undergone complete resection of a sarcoma that had arisen in the right triceps muscle. 9 months after the initial resection of the triceps sarcoma, surveillance imaging revealed a newly developed left upper lobe pulmonary mass. This lung lesion was completely resected and was a metastatic sarcoma. 9 months after the pulmonary resection, the patient presented to our institution with two episodes of acute neurologic change manifested as two brief episodes of dizziness and diplopia for 2 minutes. Subsequent imaging of the brain demonstrated two foci of acute or subacute ischemia in the superior right frontal lobe and left cerebellum, reflective of embolic lesions. Peripheral vascular imaging with carotid ultrasound did not demonstrate vascular abnormalities. Transthoracic echocardiography (TTE) revealed a large mobile mass based of the interventricular septum within the left ventricular cavity (Figure 1). Transesophageal echocardiogram (TEE) showed large mobile left ventricular sessile-appearing mass related to the interventricular septum and the subvalvular mitral apparatus (Figure 2). Finally, cardiac MRI (Figure 3) showed a mobile, oval shaped mass within the left ventricle measuring approximately 1.5 x 2.9 x 2.4 cm, in transverse anteroposterior (AP) and craniocaudal dimensions, intricately related to the chordae tendinae of the mitral valve and involving the posterior-medial papillary muscle which was continuous with, and adjacent to, the interventricular septum. The left ventricular mass was implicated by our team as the most likely source for embolic injury to the brain, and with this detailed anatomic definition, the patient was offered resection. Coronary angiography revealed no significant coronary disease. Intraoperatively, the mass appeared as a broad-based lesion originating from the tip of the posterior-medial papillary muscle and appeared to extend along the chordae tendinae toward the anterior leaflet of the mitral valve. Wide excision of the papillary muscle, the associated chordae and half of the anterior mitral leaflet, was performed in an effort to achieve negative resection margins. Initial histopathological evaluation of the resected mass revealed the lesion to be a sarcoma, and that the resection margin at the papillary muscle was positive. A wider resection of the interventricular septal tissue around the papillary muscle was performed to ultimately achieve a negative margin. Mitral valve replacement with a bovine tissue prosthesis was performed. The patient's postoperative course was complicated by atrial fibrillation and pleural effusions. She was discharged in sinus rhythm and in good health on postoperative day 10. Her histopathology was ultimately diagnosed as undifferentiated pleomorphic sarcoma (atypical spindle cell neoplasm), likely representing metastatic, rather than a primary lesion. Patient was discharged with follow-up at a regional cancer center that was originally involved with her triceps and subsequent pulmonary sarcoma resections.

## 3. Discussion

This case posed multiple diagnostic challenges that were relevant to, and ultimately defined her treatment options. Our patient presented with symptoms of a neurological event which prompted brain imaging. Cardiac tumors can be asymptomatic [13]. Our patient had involvement of her left ventricle. However, prior reports have indicated that although metastatic cells could be filtered by the pulmonary circulation, the level of vascularity may be the focus of metastases [13, 14].

Tumors occurring on the left side of the heart can produce symptoms due to direct contact with the systemic arterial circulation. Neoplastic or thrombotic emboli from the tumor nidus can lead to stroke or another systemic organ damage [9].

The driving impetus in the management of our patient was the prevention of further cerebrovascular embolic event. Given the relatively brief disease-free interval from her sarcoma diagnoses, as well as an initial surface echocardiographic finding suggestive of a large sessile left ventricular-based intracardiac mass, the initial clinical impression was to offer the patient a chemo-radiotherapy treatment option. Confidence in the ability to offer resection of the mass only occurred after further anatomical characterization of the lesion with transesophageal echocardiography and cardiac MRI. Ultimately, intraoperative analysis of the anatomical extent of the lesion mirrored our estimations from imaging, and a complete resection with negative margins was achieved. This treatment modality, in contrast to the nonsurgical treatment options that were proposed prior to anatomical delineation of the mass, offered removal of the cardioembolic nidus and the hope of completely resecting the malignancy.

For patients presenting with echogenic structures located inside the heart, multimodality imaging is often required. Initial TTE is useful; however, TEE is frequently required for comprehensive and accurate assessment [15]. The multiplanar assessment of anatomy, visualization of tissue composition with high contrast, and the functional assessment of intracardiac flow dynamics afforded by cardiac MRI provide a thorough assessment of the tumor extent and to help determine respectability. MRI allows for early differentiation between a nonneoplastic mass and a tumor mass, be it benign or malignant [16]. Although cardiac sarcomas can be a primary site, they have poor prognosis with overall survival < 1 year [17]. Prognosis is generally poor because of the role of chemotherapy may be still ill defined, and complete surgical tumor resection often proves to be challenging [18]. A contributing factor to the negative prognosis is the nature of the heart's unique anatomic and physiologic role which limits complete surgical resection of neoplastic masses. These tumors are rare, and to our knowledge, a metastatic tertiary site to the heart has not been reported.

Our case represents a rare tumor with an unusual course with three different sites involved over the clinical course of many months. It highlights the importance of multimodality imaging, a heart team approach, and oncology support to delineate a successful surgical resection and an optimal clinical result.
